# Approaches for prevention of mother-to-child transmission of HIV services during coronavirus disease 2019

**DOI:** 10.4102/hsag.v29i0.2553

**Published:** 2024-05-10

**Authors:** Livhuwani Tshivhase, Matshidiso Florence Q. Setshedi, Idah Moyo

**Affiliations:** 1 Department of Nursing, School of Health Sciences, Sefako Makgatho Health Sciences University, Pretoria, South Africa; 2 Department of HIV Services, Populations Solutions for Health, Harare, Zimbabwe; 3 Department of Health Studies, College of Human Sciences, University of South Africa, Pretoria, South Africa

**Keywords:** approaches, COVID-19, HIV, innovative, Interpretive phenomenological, PMTCT services

## Abstract

**Background:**

During the coronavirus disease 2019 (COVID-19) pandemic, there was a reduction in access to prevention of mother-to-child transmission (PMTCT) of human Immunodeficiency virus (HIV) services globally, yet this programme is critical for reducing paediatric HIV incidence. To minimise the impact of COVID-19 and prevent disruptions to the PMTCT service provision, innovative strategies had to be developed and implemented.

**Aim:**

The study aimed to describe the approaches that were developed and utilised during the COVID-19 pandemic in enhancing PMTCT services in Tshwane primary healthcare facilities.

**Settings:**

Three primary healthcare facilities that were providing PMTCT services during the COVID-19 pandemic located in the Tshwane district, Gauteng province, South Africa.

**Methods:**

The study is part of a larger study that focused on the experiences of healthcare workers who were rendering PMTCT services during the COVID-19 pandemic. An interpretative phenomenological analysis (IPA) design was employed to gain insight into the experiences of 16 purposively sampled healthcare workers who were providing PMTCT services during the pandemic in Tshwane district facilities. In-depth individual audio-recorded interviews were conducted with study participants, following a semi-structured interview guide. Data analysis was performed using an IPA framework.

**Results:**

Three superordinate themes emerged: strategies utilised for providing care, community-based initiatives, and support systems to enhance the PMTCT service access.

**Conclusion:**

Strengthening community-based initiatives and support systems is important for the enhancement of the PMTCT programme during and beyond the pandemic.

**Contribution:**

Community-based initiatives are critical in continuity of PMTCT services, reducing HIV incidence, under-five child morbidity and mortality particularly during emergency situations.

## Introduction

The prevention of mother-to-child transmission (PMTCT) of human Immunodeficiency virus (HIV) programme plays a significant role in reducing the risk of mothers transmitting HIV to their infant (Rahmadhani & Aprina 2022). Approximately 5% – 10% of HIV infections are transmitted during pregnancy, 10% – 15% during childbirth, and 5% – 20% during the breastfeeding period (World Health Organization [WHO] 2020a). According to Bisnauth et al. (2020), the PMTCT programme is considered a successful strategy that reduces the risk of mothers transmitting HIV to their infant, particularly by taking antiretroviral therapy (ART) during pregnancy and the lactation period.

The use of ART by pregnant HIV positive women is associated with reduction in the transmission of HIV infection to their unborn baby (WHO 2020b). The adherence to ART is therefore critical for the pregnant women who are diagnosed as HIV positive. The emergence of the coronavirus disease 2019 (COVID-19) lockdown impacted negatively on PMTCT services. Effective implementation of the PMTCT programme is crucial even during pandemics (UNICEF 2020).

During the COVID-19 pandemic, 60% of the chronic diseases accounted for all the deaths and HIV was one of the leading causes (Adugna, Azanaw & Sharew Melaku 2021; Mehraeen et al. 2018). At the emergence of the COVID-19 disease, focus was more on the pandemic than HIV and the Global Fund was also redirected to fund more of COVID-19 response activities (Mehraeen et al. 2020; The Global Fund 2021). Some of the people living with HIV faced financial insecurities and postponed their health appointments and discontinued ART medicines (Mehraeen et al. 2020).

In responding to the above disrupted HIV services (Mehraeen et al. 2020; Yelverton et al. 2021) telehealth was seen as a helpful method that would also help to curb COVID-19 exposure (Fodjo et al. 2020; Roldan et al., 2020). Scheduled appointment visits and clinical follow-up services were also disrupted by the pandemic, but telemedicine helped to alleviate the situation. Many countries tried several strategies to prevent disruptions to HIV service delivery, for example, sending medicines via the post office services and by extended ART refill (Ridgway et al. 2020). Other innovative strategies utilised to facilitate HIV treatment continuity and reduction of the risk of COVID-19 exposure included telehealth, broadening access to ART multi-month dispensing and concentrating on community-based case-finding, immediate initiation of ART among newly diagnosed cases (Boyd et al., 2020; Rockwell & Gilroy 2020). Evidence has also demonstrated that in a bid to increase access to HIV treatment, prevent new infections and maintain viral suppression in people living with HIV (WHO 2021), telephone consultations were employed, ART refill sites increased and there was redesigning of ART pick-up points (Fodjo et al. 2020; Rogers, Lovell & Young 2023).

Evidence has shown that during the COVID-19 pandemic communication between providers and recipients of care was mainly through health education and there were minimal counselling sessions through physical means; hence, use of virtual counselling became a key important innovation (Bisnauth et al. 2020; Okal et al. 2022). According to Okal et al. (2022) and Knettel et al. (2018), telehealth strategies became paramount in facilitating treatment continuity through sending appointment reminders and providing adherence support.

To enhance access, facilitate continuity and prevent disruption of PMTCT service delivery, innovative strategies that included the use of outreach and community-based approaches were implemented. Successes were noticed in a study by Nalubega et al. (2021) that found that these community-based outreach approaches were effectively utilised in HIV care management programmes. Other authors (Krumme et al. 2018; Grimsrud & Wilkinson 2021) also advocate for synchronisation of community clinic visits as well as use of differentiated service delivery approaches to prevent service disruption during pandemics.

The PMTCT programme implementation was also affected, just like any HIV care services by the COVID-19 pandemic. Regarding the PMTCT programme strengthening, there was a need for multi-month dispensing for long-term supply of ARV treatment and longer duration of counselling for programme sustainability, reduction of multiple visits to collect treatment and use of technology (Bisnauth et al. 2022; Izudi et al. 2022). A study by Naidoo et al. (2023) reported that more women who enrolled for antenatal care pre-COVID-19 were aware of their HIV status than those enrolled during the 2020 period. The same study also found that the HIV prevalence among women who delivered during COVID-19 period was higher than during the pre-COVID era. In Uganda, where facilities were far for HIV positive and pregnant women, ART treatment and health services were given at the comfort of their homes using a differentiated service delivery approach (the Global Fund 2021).

This study describes the innovative approaches in the prevention of mother-to-child transmission of HIV programme during the COVID-19 pandemic in healthcare facilities in the city of Tshwane, South Africa.

## Research methods and design

An interpretative phenomenological analysis (IPA) design was used for this study. This design was adopted to gain an in-depth understanding of the approaches that were used in rendering PMTCT during the COVID-19 lockdown. The design was appropriate as it offered the researchers an opportunity to dialogue with participants and gain an interpretation of the experiences from the participants’ point of view (Alase 2017; Qutoshi 2018). This design is widely used in social science research as a method to explore and interpret the lived experiences of participants from their viewpoints (Qutoshi 2018).

### Setting

The study was set in three out of the 21 primary healthcare facilities that were rendering PMTCT services during the COVID-19 lockdown in the city of Tshwane. These primary healthcare facilities offer the following services: antenatal and postnatal care, HCT (HIV counselling and testing), PMTCT, curative and chronic care, emergency care, tuberculosis (TB) treatment, immunisation, integrated child management services, family planning and emergency services. In the study setting, PMTCT services are rendered by midwives and mentor mothers who happened to be females most of the times. The facilities were purposively selected because they were high volume sites in terms of antenatal care service provision among the 21 health facilities in the city of Tshwane.

### Population and sampling

The population for this study was healthcare workers who were rendering the PMTCT services in the selected facilities during the COVID-19 lockdown. Among the healthcare workers, only nurses and lay counsellors (who were mentor mothers) were purposively selected for the study. The criteria for inclusion into the study were as follows: having worked for 6 months or more before COVID-19; having provided PMTCT services during the COVID-19 pandemic. The study excluded all healthcare providers who were not attending to PMTCT clients during COVID-19 lockdowns, those on leave during the data collection phase and those who refused to be audio-recorded. Sixteen healthcare providers constituted the sample of the study determined by data saturation.

### Data collection

Data collection commenced from 01 March 2023 to 30 April 2023. Data were collected in a private consultation room in the healthcare facility secured by the researcher for that purpose. Participants were recruited a week before actual data were collected from the selected facilities. Healthcare providers on duty took turns for interviews during their lunch times. On some days when the facility was busy, interviews were postponed for the next day. The researcher established rapport with study participants and made sure they understood the study prior to signing the informed consent form to participate in the study. Cell phone numbers were requested from the participants with an explanation that after analysis there was a need to share with each of the participants the findings for member checking processes. To ensure privacy and comfort of the participants, the researcher pasted a ‘Do not disturb’ sign on the door of the interview room. The researcher suspended her preconception when conducting interviews (bracketing). Bracketing was to enable participants to express their concerns and make claims on their own terms (Smith & Osborn 2015). Data collection was guided using a semi-structured interview guide.

The interview guide was piloted on three participants working in two of the facilities who met the inclusion criteria for the study, and their findings are not included in the study. The aim of the pilot study was to ascertain whether the questions in the interview guide were clear to participants before the actual study was conducted (Alase 2017). English was used for all participants as it is the language used when giving a report in the facilities. A voice recorder was used together with field notes to record the interviews after an explanation was carried out and the participants consented both verbally and by signing the consent. Data saturation was reached at participant number 13 and three additional participants were interviewed to confirm such. According to Saunders and Lewis (2017), data saturation is the point at which data become redundant, repetitive, or no new information emerges while collecting data from participants. The interviews lasted for 45 min to an hour with each participant.

### Data analysis

Data analysis was conducted independently by the 1st and the 3rd authors as the experienced qualitative researchers following Interpretative Phenomenological Analysis framework as outlined by Smith and Osborn (2015). The recordings were transcribed by the 2nd author who had collected the data for the study. The authors who did the coding read the transcripts more than twice to immerse themselves to the original data. When reading through the data, they wrote notes in the margins of the transcripts. Emergent themes were developed from transcripts and analysis of notes made into themes. Each author searched for connections across emergent themes and put the themes together. With each transcript reading, the authors were bracketing their preconception about the data to keep an open mind to justify individuality in each case. The researchers looked for patterns in the themes across all the cases. At this stage, the researchers moved from low-level interpretation of data to a highly detailed, interpretative, and theoretical level of interpretation of the themes as described by Smith and Osborn (2015). After this step, the authors discussed their results to see whether their interpretations were yielding similar themes and subthemes. The area with differences were then discussed until a consensus was reached. Member checking was performed telephonically (by the 2nd author who did data collection) with eight of the study participants who answered their cell phones. After the confirmation of the study’s findings, both authors discussed and reconciled the themes and finalised the table of themes. The final product of the analysis was a table of superordinate themes, themes and subthemes that were agreed upon by the authors.

### Measures to ensure trustworthiness

The researcher ensured trustworthiness in this study by adhering to credibility, confirmability, dependability, transferability, and authenticity as outlined by Gray and Grove (2023) and Bless, Higson-Smith and Sithole (2018). Credibility is the confidence of the reader about the extent to which the researcher has produced the results that reflect the views of the participants (Gray & Grove 2023). Credibility was ensured through peer debriefing wherein all the authors sat as peers and discussion on themes was performed, and a final table of themes was developed with the consensus of the peers. Member checking was carried out telephonically after analysis to ensure credibility with the study participants to verify if the findings were a true reflection of the participants’ lived experiences (Lincoln & Guba 1985). Dependability in this study was ensured by documenting detailed explanation of the methodology that was followed for the study to enable another researcher to replicate the study as articulated by Creswell and Creswell (2017). To ensure transferability, the researcher described in detail the context in which data were collected, the researcher and the relationship with the participants. Confirmability was ensured by detailed description of the methods followed detailing how and what was performed in what context so that one could replicate the study. Authenticity was ensured by keeping records as an audit trail in the locked cupboards for 5 years after the study is published and using verbal quotes in displaying the study’s findings, so the voices of participants are then heard and interpreted (Lincoln & Guba 1985).

### Ethical considerations

The study was conducted following the Helsinki Declaration principles. This was performed after receiving the ethical clearance from the Sefako Makgatho University Research Ethics Committee (SMUREC/H/281/2022:PG). Permission was also obtained from the Gauteng Department of Health, the district manager of the facilities under study and the operational manager for the facilities. All the participants voluntarily signed informed consent forms prior to the interviews. To protect the healthcare facilities, no name was mentioned. Pseudonyms were used to ensure confidentiality and anonymity.

## Results

The sample comprised 16 female participants aged between 25 years and 56 years. Table 1 presents the demographic table of the participants for this study. Of the 16 participants, 6 were mentor mothers and 10 were midwives. Fourteen of the participants were trained on PMTCT. Majority of them has over 5 years’ experience in rendering PMTCT services. Table 2 presents the summary of the themes for the study.

**TABLE 1:** Participants’ demographic data.

Number	Pseudonyms	Age (years)	Gender	Category	PMTCT training	Years of work experience (years)
01	Tshidi	53	F	Mentor mother	Yes	12
02	Alice	35	F	Registered nurse	Yes	7
03	Tshepang	25	F	Registered nurse	Yes	3
04	Thando	50	F	Registered nurse	Yes	17
05	Emma	42	F	Mentor mother	No	8
06	Angy	56	F	Registered nurse	Yes	17
07	Phathu	51	F	Registered nurse	Yes	12
08	Kganya	48	F	Registered nurse	No	4
09	Sharon	52	F	Registered nurse	Yes	10
10	Andile	54	F	Registered nurse	Yes	22
11	Sophia	38	F	Registered nurse	Yes	15
12	Lisbeth	38	F	Mentor mother	Yes	5
13	Marothi	25	F	Mentor mother	Yes	9
14	Lucky	51	F	Mentor mother	Yes	2
15	Alina	30	F	Mentor mother	Yes	5
16	Thembi	29	F	Registered nurse	Yes	4

PMTCT, prevention of mother-to-child transmission.

**TABLE 2:** Summary of superordinate themes, themes, and sub-themes.

Superordinate themes	Themes	Sub-themes
Strategies to enhance communication and continuity of the PMTCT services.	Information sharing	Health education Individual counselling
	Initiatives for PMTCT services continuity.	Telehealth initiatives Mentor-mother’s initiatives
Community-based initiatives	Ward-based outreach team (WOBOT) initiative	Tracking of referrals Maternal and child health
	Synchronisation of clinic visits	Synchronisation of PMTCT and child services Synchronisation of PMTCT and HIV or COVID-19
Support systems	Psychosocial support and follow-up services	Psychological services Nurses’ follow-up activities

PMTCT, prevention of mother-to-child transmission.

### Strategies to enhance communication and continuity of prevention of mother-to-child transmission services

This superordinate theme reflects the strategies that were utilised by healthcare providers to enhance communication and continuity of PMTCT service delivery in the context of COVID-19. Two themes emerged under this superordinate as follows: information sharing and initiatives for PMTCT service continuity.

### Information sharing

This theme is about the ways in which information was delivered to the PMTCT clients. Client-provider interaction was performed through individual counselling and health education sessions.

#### Individual counselling

Some of the clients received counselling as indicated in the following excerpts:

‘Some clients had access to counselling services through physical consultation, however the sessions were kept short as a way of preventing the spread of COVID-19.’ (Lisbeth, 38 years, mentor)

‘Because of the COVID-19 situation, we were able to follow up some clients through phone calls and or WhatsApp call after realising that they were not coming to the healthcare facility. Counselling sessions were done through these means and proved useful.’ (Alina, 30 years, mentor)

#### Health education

In the context of COVID-19, health education was the major source of communication between healthcare providers and clients, as the following excerpts show:

‘Aum, the biggest tool that we could use was giving health education. So, giving health education ensured that the mother understands the risk at hand, adhere to the treatment so that the virus is not transmitted to the baby. Some clients had access to counselling services.’ (Sharon, 38 years, nurse)

‘Health education was also the preferred method to give information to PMTCT mothers, we also try and maintain social distance as well as serve them faster.’ (Alice, 35 years, nurse)

The subtheme shared the important method of communicating with the PMTCT clients where information sharing was performed through individual counselling and health education. The counselling and the health education were for a shorter duration to limit period of exposure to COVID-19 infection.

### Initiatives for prevention of mother-to-child transmission services continuity

To facilitate continuity of PMTCT services, telehealth and mentor-mother initiatives were utilised.

#### Telehealth initiatives

Participants indicated that they had to use telehealth initiatives to facilitate PMTCT services continuity. The following extracts demonstrate this:

‘We booked our PMTCT clients on different days and did follow-up through phone calls when they miss their dates.’ (Thando, 50 years, nurse)

‘In some instances, we chatted with clients through a WhatsApp to encourage them to come to the health facility and access PMTCT services and also to provide support to the clients.’ (Tshepang, 25 years, nurse)

#### Mentor-mother initiatives

Some of the participants who were mentor-mothers working as providers of care to PMTCT clients in the selected facilities were very helpful in providing counselling, peer support and adherence to treatment. The following are their excerpts:

‘I provided them with ongoing counselling and encourage them to keep their clinic appointments as they could be given both their child immunisation and their ART in one visit.’ (Lisbeth, 38 years, mentor)

‘We provided peer support to PMTCT mothers on the importance of adhering to ARVs. We shared our lived experiences, and this was a pillar of strength for HIV positive mothers as they interacted with us.’ (Lucky, 51 years, mentor)

‘I have gone through the similar experiences, and it was easy for me to provide support to PMTCT mothers who had challenges such as breastfeeding and adherence to medication.’ (Emma, 42 years, mentor)

With the aim of improving communication with PMTCT services adherence, individual counselling and health education were adhered to, even though it was for a short duration to prevent exposure to the COVID-19 pandemic. Mentor-mothers and the telehealth were further used to promote adherence to PMTCT services.

### Community-based initiatives

This superordinate theme has two themes: Ward-based outreach team (WOBOT) initiatives and synchronisation of clinic visits. The community-based initiatives were aimed at reducing the number of clinic visits by the recipients of care as outlined next.

## Ward-based outreach team initiative

This ward-based outreach team initiatives were rendered to facilitate the tracing and tracking activities for PMTCT clients. The ward-based outreach teams were useful in tracking of referrals and maternal and child health services as outlined next.

### Tracking of referrals

The WOBOT team played a pivotal role in follow-up and tracking clients who missed clinic visits as the following excerpts show:

‘For the continuation of care on PMTCT services during the COVID-19 pandemic, the PMTCT clients were supported by members of the WOBOT. We were line listing all those that missed their scheduled visits, then submit the list to the WOBOT, that would eventually do home visits. The WOBOT gave us feedback on the outcome of their follow-up and or see clients reporting back to the clinic.’ (Angy, 56 year, nurse)

‘We would follow up the clients through a telephone, for those we failed to get, we would refer to the WOBOT. This team does the tracking and follow-up of those who miss their visits through home visits and reminding them not to miss their dates for checking their blood (PCR) from three days’ results from the hospital, ten weeks, and 14 weeks.’ (Tshidi, 53 years, mentor)

### Maternal and child health

The activities of the WOBOT also focused on enhancing maternal and child health services through providing support to pregnant and or lactating women as the following excerpts demonstrate:

‘The WOBOT checked on postnatal mothers, provided counselling, support and encouragement for example on exclusive breastfeeding for mothers that had been discharged from the hospital into the community.’ (Kganya, 48 years, nurse)

‘WOBOT also made efforts to identify pregnant women in the community, checked the booking status. They also conducted home visits during pregnancy and the postnatal period to promote healthy and safe births as well as identify danger signs. The focus was also on encouraging mothers to visit the healthcare facilities.’ (Phathu, 51 years, nurse)

## Synchronisation of clinic visits

To enhance PMTCT follow-up in the community, participants indicated that PMTCT visits were synchronised with other services. Two sub-themes emerged as follows: Synchronisation of PMTCT with other services such as child health, HIV and COVID-19 services.

### Synchronisation of prevention of mother-to-child transmission and child services

Prevention of mother-to-child transmission service appointments were scheduled at the same time with child health services as reflected in the following excerpts:

‘PMTCT services were provided in the community, at the same time with other child health services such as growth monitoring.’ (Marothi, 25 years, mentor)

‘After they had delivered their babies, we gave them appointment dates that tally with the child health immunization dates of their babies so that we can manage them concurrently.’ (Andile, 54 years, nurse)

### Synchronisation of prevention of mother-to-child transmission of HIV and coronavirus disease 2019

Prevention of mother-to-child transmission service provision was synchronised with HIV and COVID-19 activities as shown below:

‘To review PMTCT mothers in the community, review dates were put such that they would coincide with other community events such as COVID-19 vaccinations.’ (Sophia, 38 years, nurse)

‘Some clients who were bringing their babies for follow up visits were able to access their ART refills in the clinic.’ (Thembi, 29 years, nurse)

This theme outlines the main role played by the WOBOT in tracing and tracking referrals and enhancement of maternal and child health even during the COVID-19 pandemic.

The synchronisation of PMTCT services with child health, HIV and COVID-19 activities was adhered to during the pandemic to limit the number of visits to the facilities as contact was to be limited and yet adherence to PMTCT services was to be performed.

## Support systems

This superordinate theme is about the strategies that were utilised to provide support to the PMTCT clients during COVID-19. Two themes emerged as follows: psychosocial support services and follow-up services.

### Psychosocial support services

The study established that the psychosocial support was provided by psychologist.

#### Psychological services

Clients who had major psychological issues were referred to a psychologist as shown by the following excerpts:

‘Some clients had fears and anxieties about COVID-19, we had to refer them to a psychologist for expert management’ (Tshepang, 25 years, nurse)

‘We continue supporting them when they come to the clinic, they continue the counselling sessions with the psychologist as well.’ (Sharon, 52 years, nurse)

### Follow-up services

This theme is about strategies that were utilised to provide support and follow up of PMTCT clients who were already in care. Support and follow-up were conducted by both the nurses and mentor mothers through telehealth strategies such as telephones and WhatsApp.

#### Nurses’ follow-up activities

Nurses who were providing care to PMTCT clients during the COVID-19 pandemic were also supporting the adherence to PMTCT services.

The following extracts reflect the support that was provided:

‘We provided adherence and follow-up support (using telephone or WhatsApp messages) to HIV positive PMTCT mothers, mostly those with issues of concern, for example with a new HIV positive diagnosis. Some were given at least a month supply of ARVs to enhance adherence.’ (Thando, 50 years, nurse)

‘We provided counselling mainly through phone calls to address the emotional and psychosocial issues of the client. Clients expressed a lot of fears of being either pregnant or having a small baby during the COVID-19 pandemic.’ (Sophia, 38 years, nurse)

Even though mentor-mothers and WOBOT were providing adherence support to the PMTCT clients during COVID-19, nurses were mainly involved. They were the ones’ who assessed the pregnant women up to the postnatal period. They would counsel and refer the client for counselling support to the psychologist. They sought assistance of the WOBOT and the mento-mothers to follow-up the clients.

## Discussion

The aim of this study was to explore and describe the strategies that were utilised in PMTCT service delivery during the COVID-19 pandemic. Our findings show that strategies and initiatives were utilised to prevent service disruptions and to facilitate continuity of PMTCT service in the context of the COVID-19 pandemic. The discussion will focus on key findings: strategies to enhance communication and PMTCT services continuity, community-based initiatives and support systems.

This study established that in the context of COVID-19, the main source of communication that was largely health education and counselling sessions were minimal because of restrictions of contact. In a study conducted in South Africa, as in this study, Bisnauth et al. (2020) found that healthcare providers spent insufficient time on counselling and building rapport with clients receiving PMTCT services. The authors further argue that such a scenario is concerning as it has negative effects on treatment outcomes, however, this was done to curb the pandemic. According to Okal et al. (2022), counselling is a critical component of client management in HIV care and treatment services and should be patient-centred and meet the unique needs of each patient. Evidence has demonstrated that use of virtual counselling was an important initiative that was necessary to enhance PMTCT outcomes during COVID-19 pandemic (Okal et al. 2022). The use of telehealth strategies observed in this study has also proved to be effective and key to enhance the continuum of care. Knettel et al. (2018) in a systematic review for studies in Africa, demonstrated that use of telehealth in adherence counselling, reminding clients of scheduled clinic visits through phone calls and SMS text messaging proved to be effective in facilitating continuity of services.

Given the challenging restrictions related to in-person interactions during the COVID-19 pandemic, the use and expansion of virtual platforms, telephonic, SMS, WhatsApp or online follow-up are critical strategies necessary to facilitate continuity of PMTCT services (PEPFAR 2020; Vrazo et al. 2020). This study also established that health education was the major strategy for disseminating information during the COVID-19: a strategy that was highlighted as key particularly during the pandemic (UNICEF 2020).

To enhance access and continuity of PMTCT services, participants indicated that during the pandemic, services were provided through the outreach model where PMTCT follow-up visits were synchronised with community health-related services such as immunisation activities. A study in Uganda by Nalubega et al. (2021) demonstrated the effectiveness of such community-based approaches. Models such as the use of outreach approaches have proven to be effective and have been utilised in HIV care management. These interventions have been successful in routine ART management among clinically stable patients. In addition, evidence from elsewhere has shown that synchronisation programmes as an intervention improved medicine adherence for patients with cardiovascular diseases (Krumme et al. 2018). According to Grimsrud and Wilkinson (2021), in the context of COVID-19 and to prevent service disruption and improve PMTCT outcomes, use of differentiated service delivery approaches is advocated.

A study conducted in Nigeria by Sam-Agudu et al. (2017), established that the introduction of the mentor-mother initiative improved the PMTCT services delivery. Mentor mothers have been through the PMTCT programme themselves and have had similar or related experiences, hence provide peer counselling to other PMTCT clients. This study found that peer support from mentor mothers played a pivotal role in enhancing the PMTCT programme during COVID-19. Evidence has demonstrated that peer support services have been widely utilised particularly barriers that affect service uptake, adherence, and outcomes in PMTCT services (Jopling et al. 2020; Schmitz et al. 2019). In this study, mentor mothers also provided psychosocial support to PMTCT clients during the COVID-19 pandemic. These findings are like those of a study in Tanzani by Kisigo et al. (2020) where mentor mothers provide peer support in the form of adherence and psychosocial counselling. In another, study, Hamilton et al. (2020) found that HIV-positive mothers as peers in the clinic or expert patients play a significant role as a source of information and as role models particularly for the newly diagnosed HIV positive PMTCT mothers.

In the context of COVID-19, the study found that as a way of enhancing treatment continuity and provide support to mother–infant pairs, WOBOT teams utilised community outreach activities such as home visits. Similarly, the study’s findings by Ngoma-Hazemba and Ncama (2018) in Zambia, highlighted the important role played by community volunteers in conducting home visits as well as participating in outreach programmes to improve the PMTCT programme. In a related study, Suryavanshi et al. (2018) found that the PMTCT programme in India used outreach workers to do community navigation and support that resulted in enhancing uptake of services.

### Limitation of the study

The study was conducted in only three healthcare facilities situated in the Tshwane district municipality. The involvement of other districts to compare the findings might bring more understanding on the phenomenon under study. The study only focussed on healthcare providers and involving the recipients of care could have brought more understanding into the phenomenon. The recipients of care experiences could assist in developing strategies that may assist healthcare providers and recipients of care (pregnant women on PMTCT programme) to cope with future pandemics and the PMTCT programme adherence.

### Recommendations

Strengthening community-based initiatives could assist in the curbing of HIV infections to PMTCT clients. The innovations that were developed and applied during the COVID-19 pandemic could be scaled-up to improve PMTCT service provision even beyond the pandemic. These innovations could be utilised in different healthcare settings to enhance the provision of differentiated person-centredness service delivery in South Africa and in the region.

## Conclusion

Our study contributes to a growing body of evidence on the strategies and initiatives that enhances PMTCT service delivery during pandemics or a crisis. The study demonstrated that despite challenging situations related to COVID-19 restrictive measures, healthcare workers developed mechanisms to facilitate PMTCT service continuity. These findings underscore the need for strengthening and scaling-up these initiatives. The lessons learnt during COVID-19 need to be integrated into routine PMTCT service delivery in low-resource settings. If it becomes business as usual when struck by pandemics, we may lose life unnecessarily. There is a need to have policies in store for managing PMTCT services that should extend even beyond the pandemic.
